# The structural arrangement at intersubunit interfaces in homomeric kainate receptors

**DOI:** 10.1038/s41598-019-43360-x

**Published:** 2019-05-06

**Authors:** Douglas B. Litwin, Elisa Carrillo, Sana A. Shaikh, Vladimir Berka, Vasanthi Jayaraman

**Affiliations:** 1 Center for Membrane Biology, Department of Biochemistry and Molecular Biology, University of Texas Health Science Center at Houston, Houston, Texas 77030 USA; 2 MD Anderson Cancer Center UTHealth Graduate School of Biomedical Sciences, University of Texas Health Science Center at Houston, Houston, Texas 77030 USA

**Keywords:** Permeation and transport, Ion channels

## Abstract

Kainate receptors are glutamate-gated cation-selective channels involved in excitatory synaptic signaling and are known to be modulated by ions. Prior functional and structural studies suggest that the dimer interface at the agonist-binding domain plays a key role in activation, desensitization, and ion modulation in kainate receptors. Here we have used fluorescence-based methods to investigate the changes and conformational heterogeneity at these interfaces associated with the resting, antagonist-bound, active, desensitized, and ion-modulated states of the receptor. These studies show that in the presence of Na^+^ ions the interfaces exist primarily in the coupled state in the apo, antagonist-bound and activated (open channel) states. Under desensitizing conditions, the largely decoupled dimer interface at the agonist-binding domain as seen in the cryo-EM structure is one of the states observed. However, in addition to this state there are several additional states with lower levels of decoupling. Replacing Na^+^ with Cs^+^ does not alter the FRET efficiencies of the states significantly, but shifts the population to the more decoupled states in both resting and desensitized states, which can be correlated with the lower activation seen in the presence of Cs^+^.

## Introduction

Kainate receptors belong to the ionotropic glutamate receptor (iGluR) family of ion channels. iGluRs are glutamate-activated tetrameric channels canonically known for their participation in excitatory synaptic transmission and are classified into three groups, α-amino-3-hydroxy-5-methyl-4-isoxazolepropionic acid (AMPA), kainate, and *N*-methyl-d-aspartate (NMDA) receptors. Members of this family of proteins are critical for the reception of excitatory synaptic signaling, the regulation of pre-synaptic glutamate and GABA release, and the development of dendrites^1–5^. Additionally, alterations in the function of iGluRs are implicated in several pathological and disease states^5–7^. Given the important role of these receptors in physiology it becomes essential to understand all the conformational states that the receptor probes and the selection of these states associated with a given function, so that specific conformation can be targeted for specific therapeutic outcome^8^.

**Figure 1:**
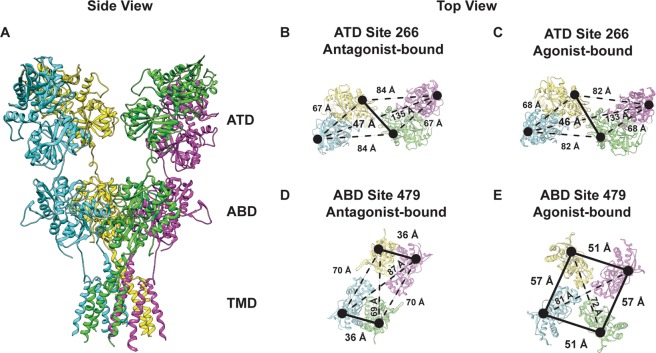
Structural arrangement of the GluK2 receptor and its FRET sites. (**A**) Cryo-EM structure of GluK2 (PDB:5KUF) showing amino-terminal domains (ATD), agonist-binding domains (ABD), and transmembrane domains (TMD). (**B**,**D**) GluK2 ATD and ABD in antagonist-bound form (PDB:5KUH). (**C**,**E**) GluK2 ATD and ABD in agonist-bound form (PDB:5KUF). Labeling sites are shown as black spheres.

Several structures are now available for the different subtypes of the glutamate receptor^9–19^. These structures show that the receptor is organized as a dimer of dimers and ligands bind to the clamshell-like agonist-binding domain (Fig. 1A). The binding of agonist induces a cleft closure conformational change which, when propagated to the transmembrane segments, is thought to lead to activation. The activated state also exhibits a coupled dimer interface at the agonist-binding domain; decoupling of the dimer interface relieves the stress on the transmembrane segments induced by the cleft closure, resulting in desensitization^9–15,18–20^. However, most of these structures are for the AMPA and NMDA subtype of the glutamate receptors. Only four full-length kainate receptor structures are available, two in the antagonist-bound form^15,21^ and two in the agonist-bound form thought to be in the desensitized state^12,15^. These structures fall into two classes, the antagonist-bound structure with tight coupling at the interfaces at both the amino-terminal domain and agonist-binding domain, and the agonist-bound structure of the kainate receptor showing large decoupling at the agonist-binding domain with a near four-fold symmetry due to extreme decoupling between the dimers (Fig. 1A,E). The closely related AMPA receptor structure exists in multiple conformations with varying degrees of decoupling at the amino-terminal and agonist-binding domain interfaces in the apo^10^ and agonist-bound forms^9–15,18,19^. Thus the question remains as to whether there is an inherent difference in the structure of kainate receptor relative to that of the AMPA receptor or is the lack of heterogeneity perceived due to the limited structural data currently available.

Kainate receptors are also unique among the glutamate receptor subtypes in exhibiting modulation by Na^+^ ions. Functional studies have shown that kainate receptors require the presence of Na^+^ and Cl^−^ ions at physiologically relevant concentrations to mediate glutamate-gated channel opening and that substitution of the sodium ions with other monovalent cations, such as cesium ions, results in the inhibition of the receptor-mediated currents^22–26^. Structural and computational studies on the isolated agonist-binding domain, as well as indirect functional studies, indicate that the dimer interface is the binding site for the Na^+ ^^20,22–28^. Given that the structural insight is based on the isolated agonist-binding domain, what is still needed to build on this foundation is insight into the modulation of the conformational and energy landscapes at this dimer interface by ions in the full-length receptor.

Recently, advances in fluorescence microscopy have made it possible to study the conformation-energy landscapes of a variety of molecules. When used in combination with previously published structural models, fluorescence resonance energy transfer (FRET) allows for the measurement of conformational heterogeneity and the energetic quantitation of dynamics within molecules^29–36^. Herein we use this methodology to study the homomeric full-length kainate receptor at sites that are able to monitor proximity within the amino-terminal domain and within the agonist-binding domain dimer interfaces that are expected to show the conformational variability associated with desensitization and ion modulation. The smFRET studies presented herein provide the first look into the structural arrangement and dynamics associated with full-length kainate receptor antagonist-binding, activation, desensitization, and sodium modulation, specifically focusing on the interfaces which are thought to be critical in these processes. One limitation of this methodology is the millisecond (5 ms bins) resolution of the method, thus rapidly fluctuating conformations will appear as an average.

## Results

### Functional characterization of FRET constructs and selection of sites

**Figure 2:**
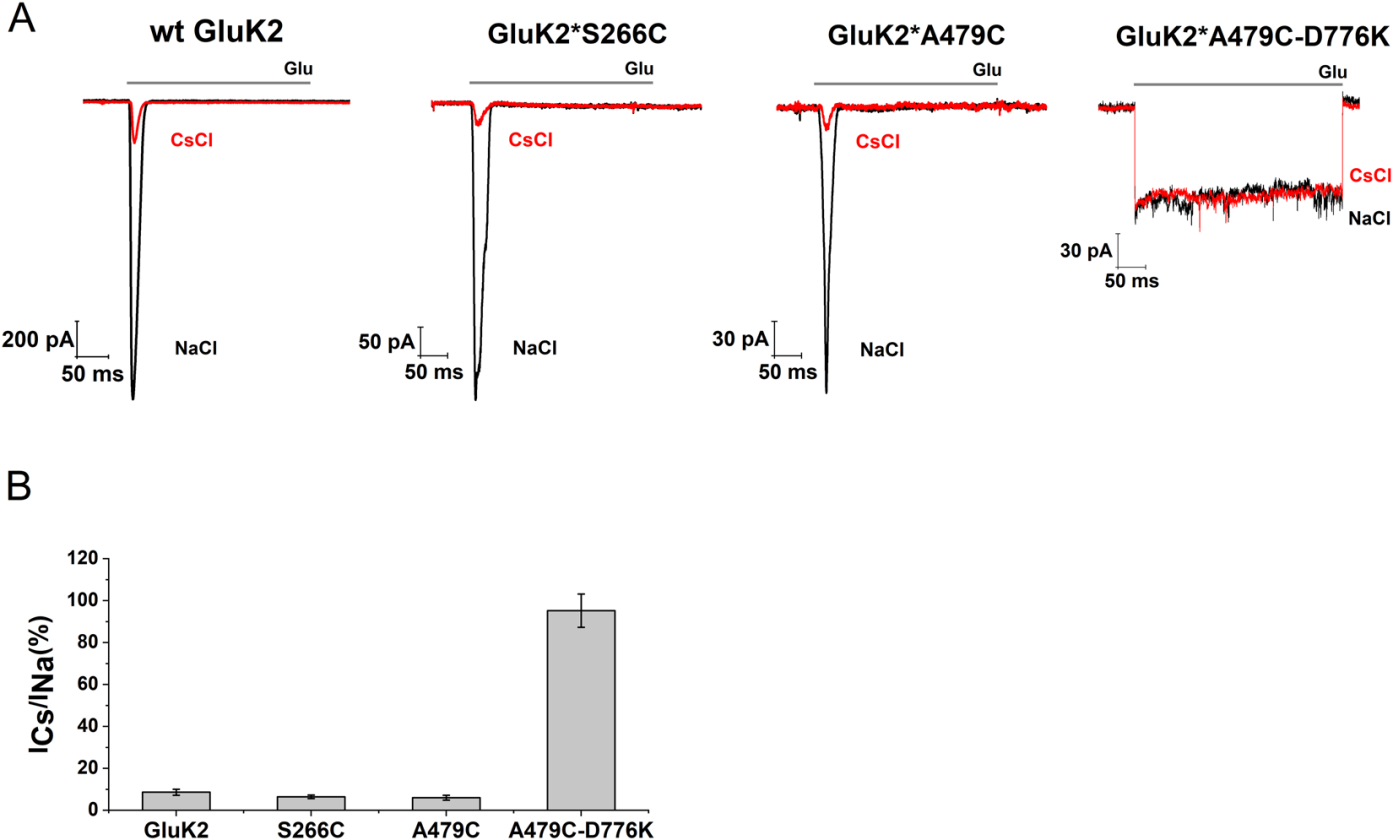
FRET construct characterization. (**A**) Representative whole-cell recordings for wild-type GluK2, and fluorophore-labeled GluK2*266C, GluK2*479 C and GluK2*479C-D776K with extracellular 150 mM NaCl (black traces) or 150 mM CsCl (red traces) and in the presence or absence of 10 mM glutamate. (**B**) Bar graph showing currents obtained using Cs^+^ buffer normalized to currents obtained using Na^+^ buffer.

The GluK2_EM_ construct used previously in cyro-electron microscopy studies^12,15^ was modified for FRET experiments to allow for site-specific labeling by mutating the non-disulfide bonded cysteines C91, C199, C432 to serines (GluK2*). Site 266 and site 479 were chosen to introduce the donor and acceptor fluorophores based on two requirements (Fig. 1). First, the sites reflected the large-scale conformational changes expected based on the currently available end state structures of the antagonist- and agonist-bound forms of the kainate receptor and closely related AMPA receptor. Second, the sites are arranged such that the distance being investigated (highlighted as darker line) has high FRET efficiency for the FRET donor-acceptor pair, all other distances are expected to have less than 15% FRET efficiency and if present should occur well separated from the distance of interest^32,35,37,38^. GluK2*-266C and GluK2*-479C constructs were characterized using electrophysiology and show kinetics similar to those of the wild type receptor (Fig. 2). Additionally, these two mutants show a similar decrease in currents upon exchange of Cs^+^ for Na^+^ in the extracellular buffer (Fig. 2).

### Conformational changes at the amino-terminal domain

**Figure 3:**
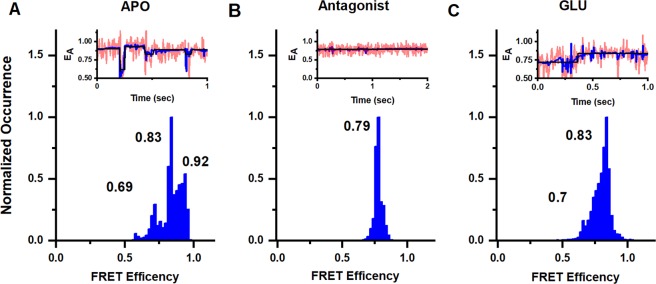
Conformational landscape of the dimer-dimer interface at the amino-terminal domain at site 266 in full length homomeric GluK2 receptors. Representative smFRET traces and FRET histograms showing fractional occurrence as a function of FRET efficiency in (**A**) the apo state (data from 47 molecules), (**B**) presence of 1 mM UBP310 (data from 29 molecules) and (**C**) presence of 1 mM glutamate (data from 50 molecules). Traces show observed signal in pink, denoised signal in blue, and state transitions in black.

Denoised smFRET efficiency histograms for the GluK2*-266C receptor and representative smFRET traces are shown for the apo, antagonist and glutamate-bound states in Fig. 3A–C, respectively. The denoised smFRET efficiency histogram was obtained using traces that exhibited single donor and single acceptor photobleaching (Supporting Fig. S1A), thus ensuring that the efficiency corresponds to a single donor-acceptor distance. Additional representative smFRET traces are provided in Supporting Fig. S2. The smFRET trajectories were analyzed using the statistical software suites STaSI^39^ (Fig. 3A–C) and HAMMY^40^ (Supporting Fig. S1B). STaSI determines the number of states within a data set using a t-test to identify step transitions and a minimum description length algorithm to determine the optimal number of states. HAMMY creates a model of the step transitions and states that best describe a data set using hidden Markov modeling. A comparison of the strengths and weakness of the two software has been preformed by Sigel and coworkers, and they showed that STaSI is more accurate in predicting number of states and state efficiencies^41^. However STaSI uses denoised data while HAMMY is performed on observed data, hence we have used both to ensure that denoising is accurate. In all cases, we see that the STaSI and HAMMY fits are in good agreement, and show the robustness of either analysis for our data.

Under apo conditions three FRET efficiency peaks are observed at 0.69, 0.83 and 0.92 corresponding to distances of 45 Å, 39 Å, and 34 Å. The smFRET histogram for the antagonist UBP-310, on the other hand, shows a single peak at 0.79 FRET efficiency indicating a single conformation, which corresponds to a distance of 41 Å. The distances of 45 Å in the apo and 41 Å in antagonist-bound state are similar to the distances of 45 Å (PDB:5WEN^19^, 5LIB^16^, 3KG2^9^, 4U4G^13^) and 42 Å (PDB:4U2P^10^) at equivalent sites seen in the antagonist and apo state structures of closely related AMPA receptors. In the glutamate-bound state two FRET efficiency peaks are observed at 0.7 and 0.83 that correspond to distances 44 Å and 39 Å. These distances are similar to the 46 Å (PDB:5VHZ^18^, 4U4F^13^) and 40 Å (PDB:4U2Q^10^) seen in the glutamate-bound structures of closely related AMPA structures, and 46 Å in the agonist-bound structure of kainate receptors (PDB:4UQQ^12^). The spread of states observed in the amino-terminal domain in the glutamate-bound state of kainate receptors do not show large decoupling as seen in Class II and Class III agonist-bound structures of AMPA receptors^15^.

### Conformational changes at the agonist-binding domain

**Figure 4:**
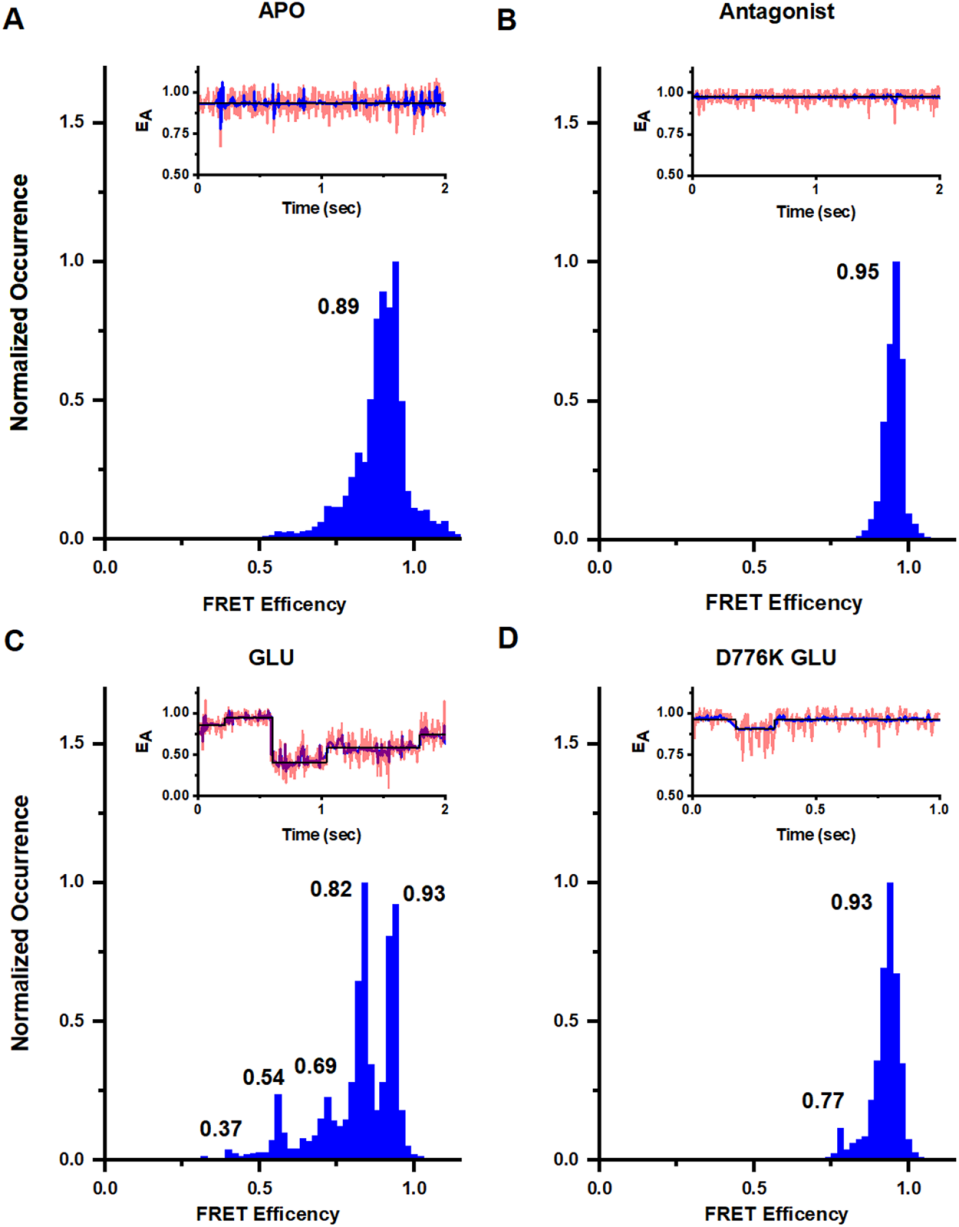
Conformational landscape at dimer interface at the agonist-binding domain at site 479 in full length homomeric GluK2 receptors. Representative smFRET traces and FRET histograms showing fractional occurrence as a function of FRET efficiency in presence of 150 mM NaCl in the (**A**) apo state (data from 57 molecules), (**B**) presence of 1 mM UBP310 (data from 28 molecules), and (**C**) presence of 1 mM glutamate (data from 66 molecules), and (**D**) D776K mutant in the presence of 1 mM glutamate (data from 47 molecules).

To characterize the conformational and energy landscape at the dimer interface of the agonist-binding domain, smFRET experiments were conducted using GluK2*-479C. smFRET histograms for mutant GluK2*-479C in the apo, antagonist, and glutamate-bound states in the presence of Na^+^ are shown in Fig. 4A–C. Additional representative smFRET traces are provided in Supporting Fig. S3 and corresponding HAMMY fits for the data are show in in Fig. S1C. The apo state in the presence of Na^+^ has a single peak in histogram showing a single state with a FRET efficiency of 0.89, corresponding to a distance of 36 Å. The antagonist-bound state also shows a single peak but with a more narrow halfwidth indicating a more rigid protein. The FRET efficiency is also higher 0.95 corresponding to a distance of 31 Å. These data are representative of a coupled agonist-binding domain dimer interface and similar to the 33 Å seen in the antagonist-bound structure of kainate receptor (PDB:5KUH^15^).

The smFRET histogram in the presence of glutamate and Na^+^, on the other hand, shows five FRET efficiencies, (Fig. 4C), corresponding to distances of 55 Å, 49 Å, 44 Å, 39 Å and 33 Å. These distances are similar to the two distances of 57 Å and 51 Å between the dimers in current kainate receptor models (PDB:4UQQ^12^, 5KUF^15^). However, a large fraction of the receptors show a less decoupled interface and these shorter distances have been observed in AMPA receptors, with structures showing a distance of 46 Å (PDB:5VHZ^18^, 5VOV^17^) and a distance of 39 Å (PDB:4U2Q^10^, 4U4F^13^) at the equivalent sites.

smFRET was next used to characterize the active state structural dynamics of the kainate receptor using a D776K mutant, which stabilizes the receptor in the open state^42^ (Fig. 4D). Additional representative smFRET traces are provided in Supporting Fig. S4. The smFRET data show that for this open-state stabilized receptor, the primary state has a FRET efficiency of 0.93, which corresponds to a distance of 33 Å. While both the apo state and the D776K glutamate-bound state have primarily one main conformation, the striking difference between the two is the half width of these states. The half width is narrower in the D776K glutamate-bound state relative to that in the apo state of the receptor, indicating that the protein is more rigid in the D776K glutamate-bound state relative to the apo state of the receptor.

### Conformational modulation by ions

**Figure 5:**
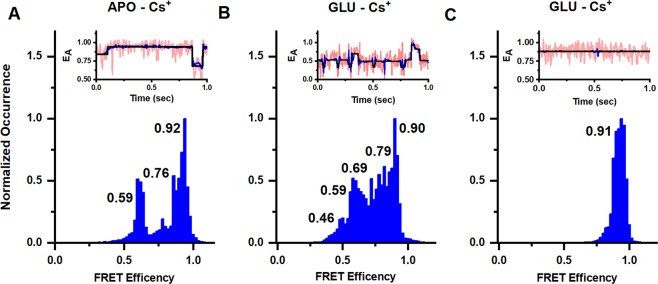
Conformational landscape of the dimer interface at the agonist-binding domain at site 479 in full length homomeric GluK2 receptors. Representative smFRET traces and FRET histograms showing fractional occurrence as a function of FRET efficiency in presence of 150 mM CsCl in the (**A**) apo state (data from 55 molecules), (**B**) presence of 1 mM glutamate (data from 52 molecules), and (**C**) D776K mutant in the presence of 1 mM glutamate (data from 55 molecules).

To study the structural effects of ion modulation, smFRET measurements were performed with the kainate receptor in the presence of Cs^+^ ions (replacing the Na^+^ ions). Cs^+^ was chosen as there are extensive electrophysiological studies performed under these conditions showing large decreases in currents^22–25^. Thus, direct correlations can be made between the smFRET data and this large body of functional studies. Changes are observed between the Na^+^ and Cs^+^ conditions in the apo state of the receptor. The smFRET denoised histograms show three efficiency peaks in the presence of Cs^+^ (Fig. 5A) corresponding to distances of 48 Å, 41 Å, and 34 Å, respectively. Additional representative smFRET traces are provided in Supporting Fig. S5. The Cs^+^ conditions are in contrast to the apo state of the receptor in the presence of Na^+^, where the receptor exists primarily in the high FRET more coupled state. The smFRET denoised traces for the glutamate-bound state in the presence of Cs^+^ (Fig. 5B) showed FRET efficiencies corresponding to five distances of 51 Å, 48 Å, 44 Å, 40 Å, and 35 Å. These distances in Cs^+^ are similar to the distances of 55 Å, 49 Å, 44 Å, 39 Å and 33 Å observed in Na^+^. However, the occupancies of these states are significantly different, with higher occupancy of lower FRET states in Cs^+^ relative to Na^+^. These results indicate that while the receptor occupies similar conformational states in both Cs^+^ and Na^+^, the more decoupled states have higher occupancy in the presence of Cs^+^.

The D667K mutant on the other hand did not show any significant shift in the states between Na^+^ (Fig. 4D) and Cs^+^ (Fig. 5C). Given that the activated state requires coupling between the dimers, the larger fraction of the receptors in the low FRET decoupled states in the apo state of the receptor in the presence of Cs^+^ would contribute to the decrease in activation observed in the presence of Cs^+^.

### State transitions and energy landscape

**Figure 6:**
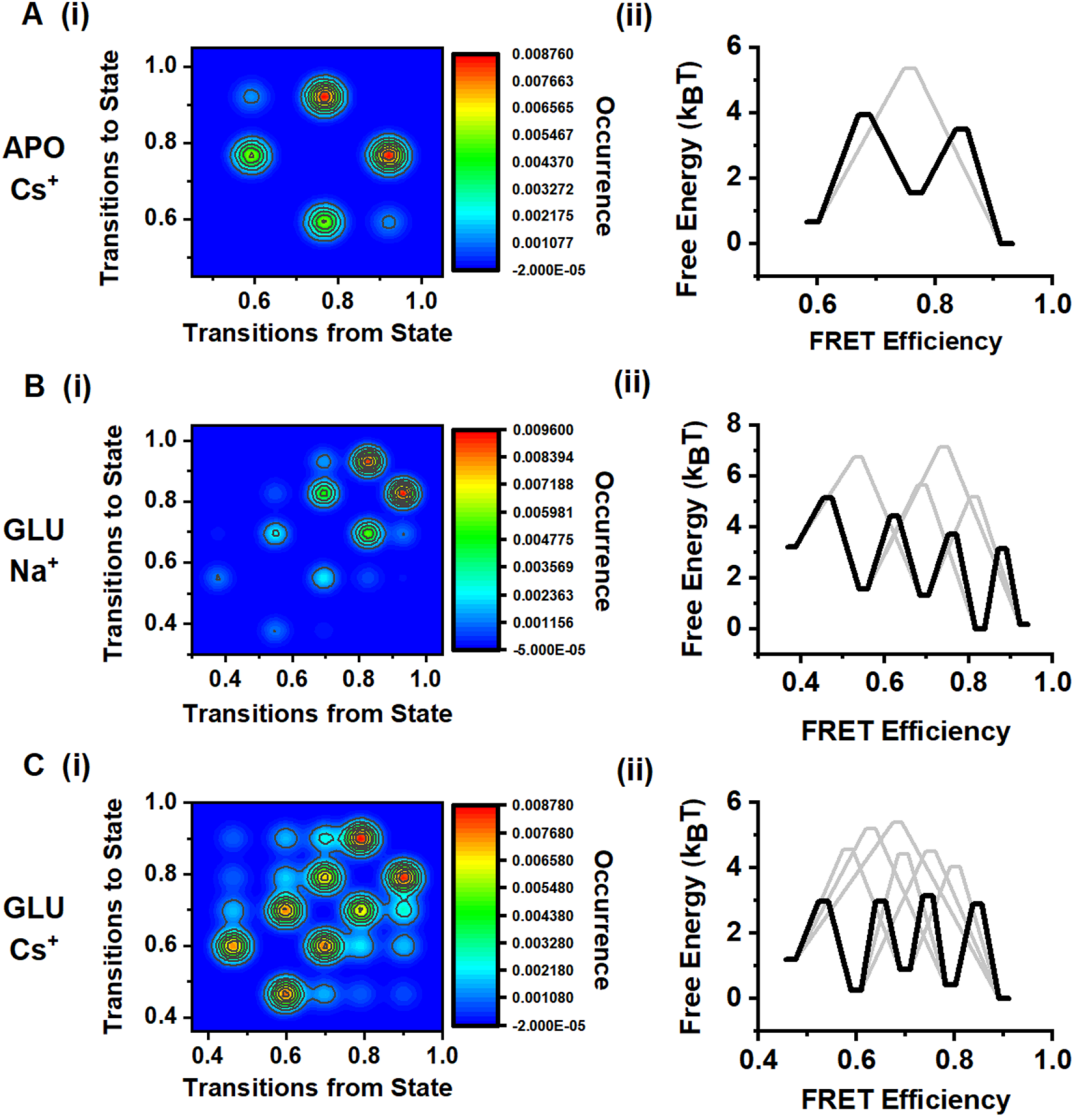
Transition maps and free energy diagrams based on the smFRET data at site 479. Transition maps showing transitions from one FRET efficiency to another FRET efficiency (i) and free energy associated with the transitions (ii) in (**A**) 150 mM CsCl, (**B**) 150 mM NaCl and 1 mM glutamate, and (**C**) 150 mM CsCl and 1 mM glutamate.

In addition to providing the state occupancy, the smFRET trajectories allow for the direct observation of transitions between different states, specifically in the desensitized and Cs^+^ conditions, as exemplified by the representative traces in Figs 4 and 5. Based on these data, we have obtained transition maps showing the relative number of transitions between states (Fig. 6). The data show that within the agonist-binding domain, transitions primarily occur between states of nearest FRET efficiency, whereas transitions between non-adjacent states are less common. The probability of observing these non-adjacent state transitions is higher in the presence of Cs^+^ under desensitizing conditions, which suggests a lower energy barrier for the transitions. These data are consistent with the energy maps and show that in the presence of both glutamate and Na^+^, the lowest energy barrier of transition is between the high-FRET states with efficiencies of 0.93 and 0.82. However, in the presence of both glutamate and Cs^+^, the activation energy barriers are similar across all states. These data indicate that with Na^+^ present the receptor is more stable in the coupled state and requires more energy to transition into states with increasing distance, and that in the presence of Cs^+^ the receptor is able to move across states with relatively low energy barriers.

## Discussion

There are several X-ray crystallographic structures of isolated domains for all subtypes of the ionotropic glutamate receptor. Studies of the full-length receptor, however, have been primarily focused on the AMPA and NMDA subtypes^9–12,14,15,18^, with only an antagonist- and agonist-bound structure of the full-length kainate receptor. The structures^9–15,18,19^, spectroscopic investigations^29,30,32,34,36,43,44^, and molecular dynamic simulations of AMPA and NMDA receptors^8,45,46^ show that the protein occupies multiple conformations under any given condition and such diversity in conformation is consistent with the diversity of states as seen in single channel recordings of these receptors. More importantly, these studies suggest that the receptor function is dictated to a large extent by conformational selection. However, this structural heterogeneity has not been shown for the kainate receptors. Herein we have use smFRET measurements to resolve the conformational landscape of the full-length kainate receptor under physiologically-relevant conditions in the antagonist-bound, apo, active, and desensitized states and additionally have identified the changes in kainate receptor dynamics induced by Na^+^ modulation.

### The resting state

Detailed structural models of the apo state of the kainate receptor have been particularly elusive; the only structural information with respect to the resting state has been derived from the structure of the antagonist-bound form of the receptor. The smFRET data show that under apo conditions in the presence of Na^+^, the agonist-binding domain exists primarily in one conformation, with a distance consistent with a coupled dimer interface, but structural heterogeneity is observed at the amino-terminal domain with a small distance range. This is similar to what is seen in the AMPA receptors^19^. In the presence of Cs^+^ ions, on the other hand, the dimer interface exhibits both coupled and decoupled conformations. Because the active state requires the agonist-binding domain interface to be coupled, this decoupled dimer interface would require more energy to be converted to the active state and can account for the lower receptor activation observed in the presence of Cs^+^.

### The antagonist-bound state

The smFRET histograms for the antagonist-bound state at both the amino-terminal domain and agonist-binding domain show single states that are similar to the most probable state seen in the resting apo state. However, the antagonist-bound state is clearly more rigid at these interfaces, exhibiting a smaller halfwidth even in this single state.

### The active state

The smFRET data on the D776K mutant, which stabilizes the receptor in the open, activated state, show that the agonist-binding domain dimers remain primarily coupled in the active state and that substituting Cs^+^ for Na^+^ has no effect. These data are consistent with the structure and molecular dynamics simulations of the D776K mutant that show the introduced lysine can occupy the Na^+^ binding site^20^, eliminating the requirement for Na^+^ to activate. Furthermore, these data are consistent with the electrophysiological measurements which show a similar extent of activation under both Na^+^ and Cs^+^ conditions^20^.The smFRET data showing a tightly coupled active state in the D667K mutant with a much smaller full width at half maximum relative to the apo state, indicates that this structural rigidity at the interface allows for the channel to be constitutively active with high probability of opening as seen in single channel recordings^20^.

### The desensitized state

Current structures of kainate and AMPA receptors under desensitizing conditions show significant differences between the two closely-related subtypes. At the amino-terminal domain, the kainate receptor showed minimal decoupling, while the AMPA receptor showed varying degrees of decoupling. At the agonist-binding domain the kainate receptors showed complete decoupling with the receptor transitioning into a near four fold symmetry, while the AMPA receptor showed smaller decoupling^9,10,13,14,18^. However cysteine crosslinking studies by Soblovesky and coworkers question the large decoupling seen in the AMPA receptors^47^, as desensitization is observed even in the cross-linked non-decoupled receptor.

The smFRET measurements under desensitizing conditions showed heterogeneity at the dimer-dimer interface at the amino-terminal domain and minimal decoupling. This would be similar to the non-decoupled states seen in with crosslinking^47^. Additionally, the smFRET data show that the large decoupled agonist-binding domain structure seen in the cryo-EM structure accounts for a small fraction of the receptors in the desensitized state. However, a large fraction of the receptors show no or slight decoupling similar to what has been observed in the AMPA receptors. Based on the smFRET measurements it can be concluded that kainate and AMPA receptors exhibit largely similar trends in terms of structural heterogenity in the desensitized state with much smaller differences then was previously thought.

smFRET measurements characterizing the proximity of the agonist-binding domain dimers in the presence of Cs^+^ show that the more decoupled states are favored, and the energy barrier is lowered for transitions to the more decoupled states. This decrease in energy barrier is consistent with MD simulations that showed a decrease in the work required to decouple the dimer interface in the absence of Na^+^ ions^20,28^. The fact that the conformational states of the agonist-binding domain are the same in both Na^+^ and Cs^+^ conditions suggest a conformational selection mechanism. The occupancy of similar states is consistent with the fact that the X-ray structures of the isolated agonist binding domain are similar under both Na^+^ and Cs^+^ conditions. The smFRET data adds to this prior knowledge by showing that the occupancy of these states are shifted.

## Conclusions

Using smFRET measurements we have characterized the conformation and energy landscapes of the kainate receptor in the apo, antagonist-bound, active, desensitized, and Na^+^-modulated states. These data suggest a similar conformational heterogeneity as seen in the AMPA receptors. The desensitized and resting states of the receptor are energetically altered in the presence of Cs^+^, which drives the protein into a decoupled dimer state which in turn leads to lower activation.

## Methods

### Generation of FRET constructs

The *R. norvegicus* GluK2_EM_ construct^12,15^ was kindly provided by Dr. Mark Mayer and retained the native glutamine at site 590. The coding sequence for GluK2 was PCR amplified and inserted into pcDNA3.1. Mutations were introduced using standard PCR-based mutagenesis methods. To create the background construct GluK2*, non-disulfide-bonded cysteines at sites C91, C199, C432, were mutated to serines. On this background two constructs were made; one with S266 mutated to cysteine and one with A479 mutated to cysteine. A third construct was made in which both A479C and D776K mutations were introduced.

### Electrophysiology

HEK 293 T cells were transfected using jetPRIME PolyPlus (wt-GluK2, GluK2*S266C, and GluK2*A479C) or lipofectamine 2000 Invitrogen (GluK2*A479C-D776K), and were, in both conditions, co-transfected with GFP at a microgram ratio of 3:1 per 10 ml of media. After 4–6 h of incubation, cells were re-plated (30 mm dishes) at low density. Cells were labeled in dish with 400 nM of donor fluorophore Alexa 555 maleimide (ThermoFisher) and 400 nM of acceptor fluorophore Alexa 647 maleimide (ThermoFisher) in 2 mL extracellular buffer pH 7.4 (150 mM NaCl, 1.8 mM MgCl_2_, 1 mM CaCl_2_, 3 mM KCl, 10 mM glucose, and 10 mM HEPES). Whole cell patch clamp recordings were performed 24–48 h after transfection, using fire-polished borosilicate glass (Sutter instruments) pipettes with 3–5 mΩ resistance, filled with internal solution: 110 mM CsF, 30 mM CsCl, 4 mM NaCl, 0.5 mM CaCl_2_, 10 mM HEPES, and 5 mM EGTA (adjusted to pH 7.4 with CsOH). The external solutions containing 150 mM NaCl or CsCl, 2.8 mM KCl, 1.8 mM CaCl_2_, 1.0 mM MgCl_2_, and 10 mM HEPES (adjusted to pH 7.4 with NaOH or CsOH) were (without and with 10 mM glutamate) applied to lifted cells using a stepper motor system (SF-77B; Warner Instruments) with triple barrel tubing. Recordings were performed using an Axopatch 200B amplifier (Molecular Devices) at −60 mV hold potential, acquired at 10 kHz using pCLAMP10 software (Molecular Devices), and filtered online at 5 kHz.

### smFRET sample preparation

HEK293T cells were transiently transfected according to JetPrime protocol at 10 µg per 10 cm plate. One day post-transfection, cells from two 10-cm dishes were harvested and washed with extracellular buffer and labeled for 1 h at room temperature with 400 nM of donor fluorophore Alexa 555 maleimide (ThermoFisher) and 400 nM of acceptor fluorophore Alexa 647 maleimide (ThermoFisher) in 3 mL extracellular buffer. This concentration of fluorophore was determined to be optimal for single donor and single acceptor labeling. After washing, labeled cells were then solubilized for 1 h at 4 °C in solubilization buffer consisting of phosphate-buffered saline, 1% lauryl maltose neopentyl glycol (Anatrace), 2 mM cholesteryl hydrogen succinate (MP Biomedicals), and ¼ protease inhibitor tablet (Pierce). Unsolubilized debris were then spun down for 1 h at 100,000 × g at 4 °C, and the supernatant used as the smFRET sample. Samples were then diluted 1:2 in cold SB before application.

### smFRET flow chamber preparation

Coverslips (22 × 22 mm No. 1) were washed with Liqui-Nox phosphate-free detergent (Alconox Inc.) and 4.3% NH_4_OH and 4.3% H_2_O_2_. Coverslips were then plasma cleaned using a Harrick Plasma PDC-32G Plasma Cleaner and then aminosilanized through Vectabond treatment (Vector Laboratories). A circular area on the slide was then isolated using Silicone templates (Grace bio-Labs) and treated with a PEG solution containing 5 kDa biotin-terminated PEG (2.5% w/w in molecular biology grade (MB) water, NOF Corp.), and 5 kDa mPEG succinimidyl carbonate (25% w/w in MB water, Laysan Bio Inc.) in 0.1 M sodium bicarbonate (Sigma-Aldrich) overnight in a dark and moist environment. On the day of the experiment, the coverslips were washed with PBS, treated with short chain 333 Da NHS-ester PEG (Thermo Scientific) and incubated for 2–3 h. Slides were then washed and dried with N_2_ gas. A chamber was then constructed over the treated circular area by applying hybriwell chambers (Grace bio-Labs) then dual silicon press-fit tubing connectors (Grace bio-Labs).

### smFRET protein preparation and attachment to coverslips

Streptavidin was applied to the chamber by flowing 32 µl of a buffer solution containing phosphate-buffered saline (PBS), 1 mM DDM (*n*-dodecyl-β-D-maltoside), 0.2 mM CHS (cholesteryl hydrogen succinate), and 0.2 mg/mL Streptavidin through the flow chamber and incubating for 10 min. 10 nM of biotinylated goat Anti-Rabbit IgG (H + L) secondary antibody (Jackson Immunoresearch Laboratories, Inc., cat. no. 111-065-003) was then flowed into the chamber, incubated for 20–30 min, then washed with PBS. Next, 10 nM of anti-GluK2 (C-terminal epitope) mouse monoclonal primary antibody (Abcam, cat. no. ab15307) was flowed in, incubated for 20–30 min, and washed with PBS. This antibody was chosen as it is far from the extracellular sites being studied. Detergent solubilized HEK293T cell membranes containing GluK2 receptors were then bound to a glass slide using the *in situ* immuno-precipitation (SiMPull^48^) method for FRET data acquisition. 60 μL of the sample was applied twice through the chamber followed by a 20–30 min incubation before flushing the chamber with 60 µl oxygen scavenging solution buffer system (ROXS) buffer twice. ROXS buffer used consisted of 1 mM methyl viologen, 1 mM ascorbic acid, 0.01% w/w pyranose oxidase, 0.001% w/v catalase, 3.3% w/w glucose (all from Sigma-Aldrich), 1 mM DDM (Chem-Impex), and 0.2 mM CHS (MP Biomedicals, LLC) in PBS, pH 7.4. 1 mM glutamate was added and/or 150 mM CsCl was used to replace NaCl in the ROXS to achieve the experimental conditions.1 mM UBP310 in the ROXS buffer was included for antagonist-bound experiments.

### smFRET data acquisition

Single molecule FRET measurements were acquired using a custom-built PicoQuant MicroTime 200 Fluorescence Lifetime Microscope. smFRET data acquisitions were conducted using pulsed interleaved excitation at 80 MHz. Both 532 nm (LDH-D-TA-530; Picoquant) and 637 nm (LDH-D-C-640; Picoquant) lasers were simultaneously used to characterize the fluorescent behavior of both fluorophores and the efficiency of energy transfer between molecules potentially showing FRET. The sample slide was immobilized on a scanning *x*-*y*-*z* piezo stage (P-733.2CD; Physik Instrumente) while being excited and observed through a 100x oil immersed lens (100 × 1.4 NA; Olympus). The photons emitted from the sample post-excitation were collected back through the objective, separated through a dual band dichroic beam splitter (Zt532/640rpc-UF3; AHF/Chroma) and sent to two SPAD photodiodes (SPCM CD3516H; Excelitas technologies) preceded by excitation filters. A 550 nm (FF01-582/64;AHF/Semrock) and 650 nm (2XH690/70;AHF) emission filter were used for the donor and acceptor channels, respectively. All acquisitions were performed in the presence of a photo-stabilizer and oxygen scavenging solution buffer system (ROXS).

### smFRET molecule selection and analysis

Since the kainate receptors studied here are homomeric, there is a distribution of various donor/acceptor combinations. To exclude signal from those channels having multiple donors or multiple acceptors, the fluorescence intensity of single channels and the step-wise photobleaching was studied. Multiple donors or acceptors have multiple photobleaching steps and these traces were not used. The number of photobleaching steps per molecule in the 266 and 479 data sets exhibited the following distribution: 10% showed four steps, 50% showed three steps, 35% showed two steps, and 5% showed one step. The FRETing regions of the smFRET traces obtained for all constructs were on average 1 to 3 seconds in length. Only the traces with a clear single photobleaching step in both donor and acceptor channels were included in the analysis. This molecule hence reports on a single distance between single donor and single acceptor. Such a strategy has been used successfully by us as well as several other laboratories^35,36,49,50^.

The fluorescence intensity of the donor and acceptor (upon excitation of donor) were used to calculate FRET efficiencies as described in these references^29–36^. The photon counts produced per donor and acceptor excitation were acquired at 1 ms resolution, binned to 5 ms, and denoised with wavelet decomposition, and the calculated efficiencies were then plotted as separate histograms showing the occurrence of photons at their observed FRET efficiencies. Each count in the histogram represents one 5 ms bin, with the cumulative of all such counts from all the molecules normalized.

The number of states that best describes the distribution of FRET efficiencies found in the obtained FRET data was then determined using Step Transition and State Identification (STaSI) analysis^39^ and hidden Markov modeling using HAMMY^40^ and fit to Gaussian distributions.

While we have provided distances based on the smFRET intensities it should be noted the distances are between the fluorophores and hence the size and length of the fluorescent probes brings additional errors in the estimation of distances. Thus we focus on the change in distances and heterogeneity between the different states being studied. This is a reasonable assumption as the size of the probes is not expected to change between the states being studied. Furthermore, the heterogeneity and transitions across states are evident in the single molecule traces where a given donor-acceptor pair is being probed.

### Free energy calculations

The free energy of the most populated state identified by STaSI analysis was set to 0 *k*B*T*. The percent occupancies as determined by STaSI were then used to calculate the equilibrium constant *K*_eq_ between states, and the free energy of every state relative to the most populated state was determined using the equation:

$${\rm{\Delta }}{G}^{0}=-\,{k}_{{\rm{B}}}{T}\,\mathrm{ln}\,{{K}}_{{\rm{eq}}}$$

The transition probabilities between each pair of states, given our 5 ms bin time, was used to determine the reaction rate for each transition, and the heights of the energy of activation barriers were calculated assuming a first-order reaction rate and using the Arrhenius equation:

$$k=A{e}^{-Ea/{k}{\rm{B}}{T}}$$

where k is the rate constant, the concentration of the starting state was taken as the STaSI-derived fractional occupancy of that state, and the value of the pre-exponential was chosen to be 10 ms^−1^. Forward and reverse energies of activation were averaged in the final figure.

### Statistics

Data were analyzed using Origin (OriginLab Corp.), MATLAB (MathWorks), and Excel (MicrosoftCorp.). For smFRET experiments, the numbers of molecules that passed cross- and anti-correlation checks for A479C-apo, A479C-glutamate, A479C-apo-CsCl, A479C-glutamate–CsCl, S266C-apo, S266C-glu, D776K-glutamate, D776K-glutamate- CsCl, S266C-UBP310 and A479C-UBP310 were, respectively, *n* = 57, 66, 55, 52, 47, 50, 47, 55, 29 and 28.

## Supplementary information

Supplementary information

## Data Availability

The datasets generated during and/or analyzed during the current study are available from the corresponding author on reasonable request.
