# Supplementary material for: The structural arrangement at intersubunit interfaces in homomeric kainate receptors
Source: Sci Rep. 2019 May 6;9:6969. doi: 10.1038/s41598-019-43360-x (PMC6502836; doi:10.1038/s41598-019-43360-x)
Supplement: Supplementary file 1 — Supplementary information [file 41598_2019_43360_MOESM1_ESM.pdf]

The structural arrangement at intersubunit interfaces in homomeric kainate receptors

Douglas B. Litwin[1,2], Elisa Carrillo[1], Sana A. Shaikh[1], Vladimir Berka[1], and Vasanthi Jayaraman[1]*

[1] Center for Membrane Biology, Department of Biochemistry and Molecular Biology, University of Texas Health Science Center at Houston, Houston, Texas 77030, USA.[2] MD Anderson Cancer Center UTHealth Graduate School of Biomedical Sciences, University of Texas Health Science Center at Houston, Houston, Texas, 77030, USA.

*To whom correspondence should be addressed: Vasanthi Jayaraman, Department of Biochemistry and Molecular Biology, Center for Membrane Biology, University of Texas Health Science Center, MSB 6.174, 6431 Fannin St, Houston, Texas 77030; email: vasanthi.jayaraman@uth.tmc.edu; Tel.: (713) 500-6236.

Supporting information:

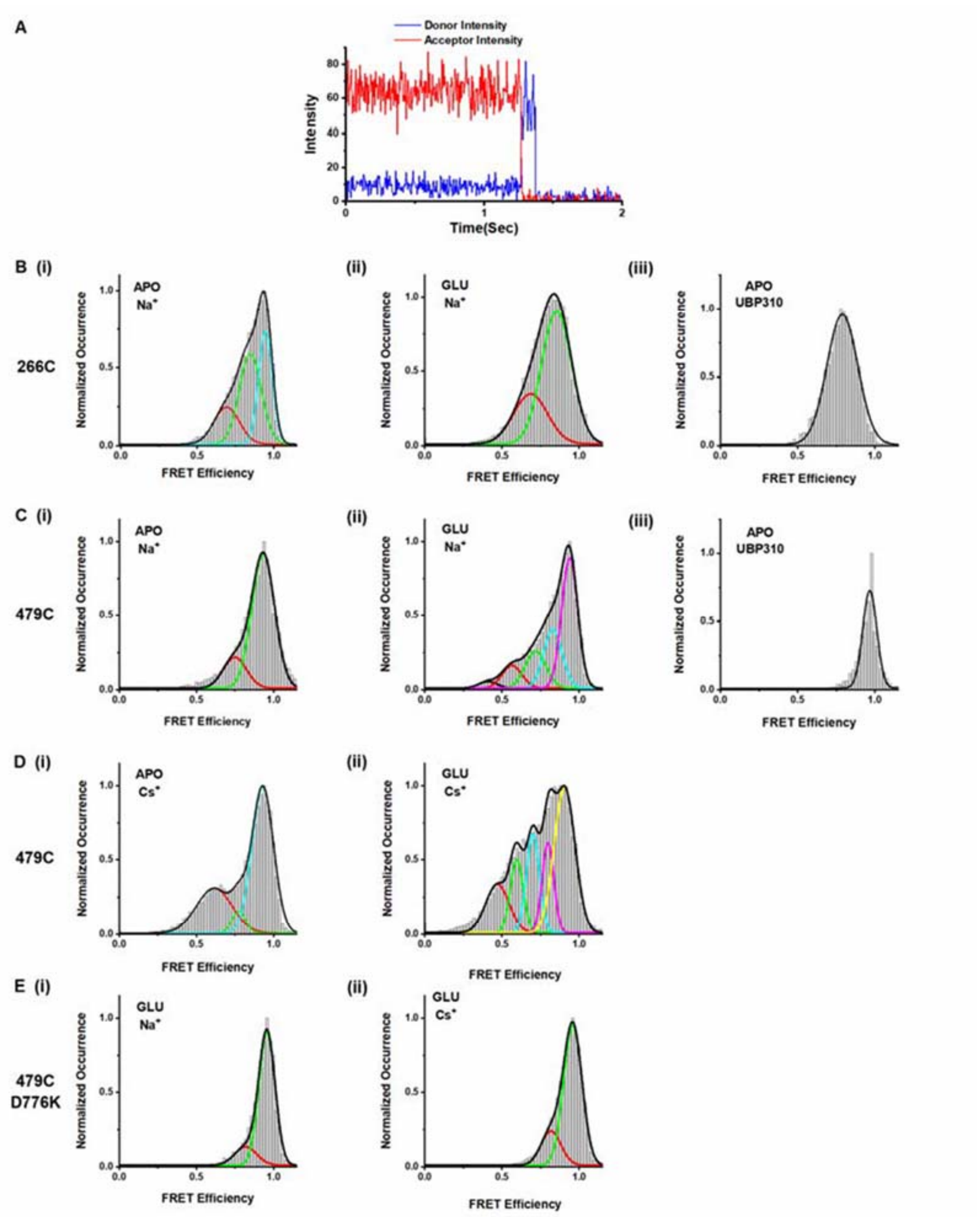

Figure S1:
(A) Representative traces showing single photobleaching step for donor and acceptor and anticorrelation showing that the donor and acceptor are from FRET pairs. Observed histograms with data being fit using Gaussians based on HAMMY analysis for (B) GluK2*S266C (i) in apo conditions, (ii) in the presence of 1mM glutamate and (iii) in the presence of 1 mM UBP310. (C) GluK2*479C with 160 mM NaCl and in apo conditions (i), (ii) in the presence of 1mM glutamate and (iii) in 160 mM NaCl with 1mM UBP310. (D) GluK2*479C with 150 mM CsCl and in the absence (i) and presence (ii) of 1mM glutamate.(E) GluK2*479C-D776K with (i) 160 mM NaCl and 1mM glutamate and (ii) 150 mM CsCl and 1mM glutamate.

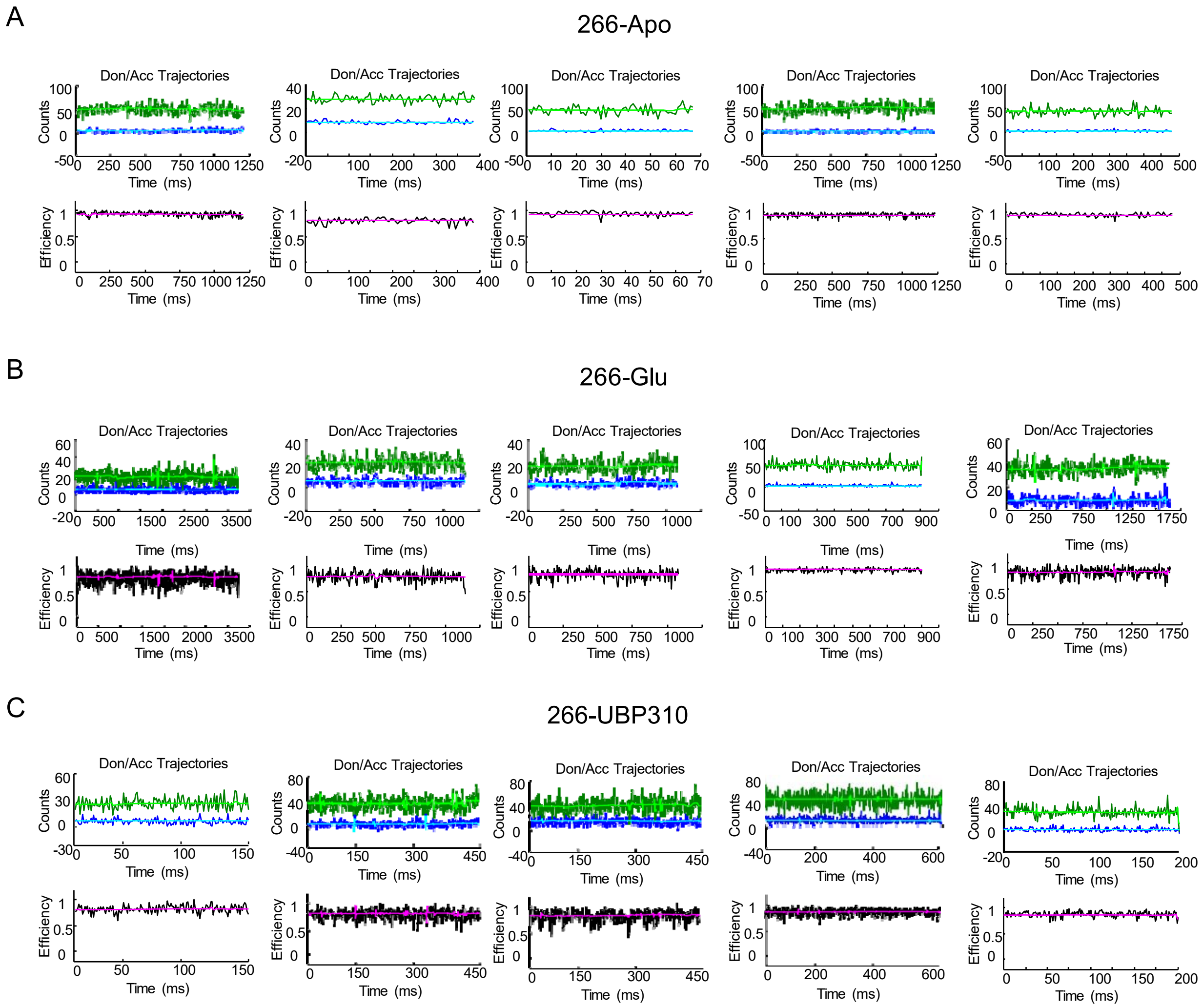

Figure S2

Representative FRET efficiency traces showing the donor (blue), acceptor (green) and FRET efficiency (black) with wavelet denoising shown in cyan, neon green, and magenta respectively, in the FRET region for site 266 in the (A) apo state, (B) glutamate-bound state, and (C) UBP310 antagonist bound states.

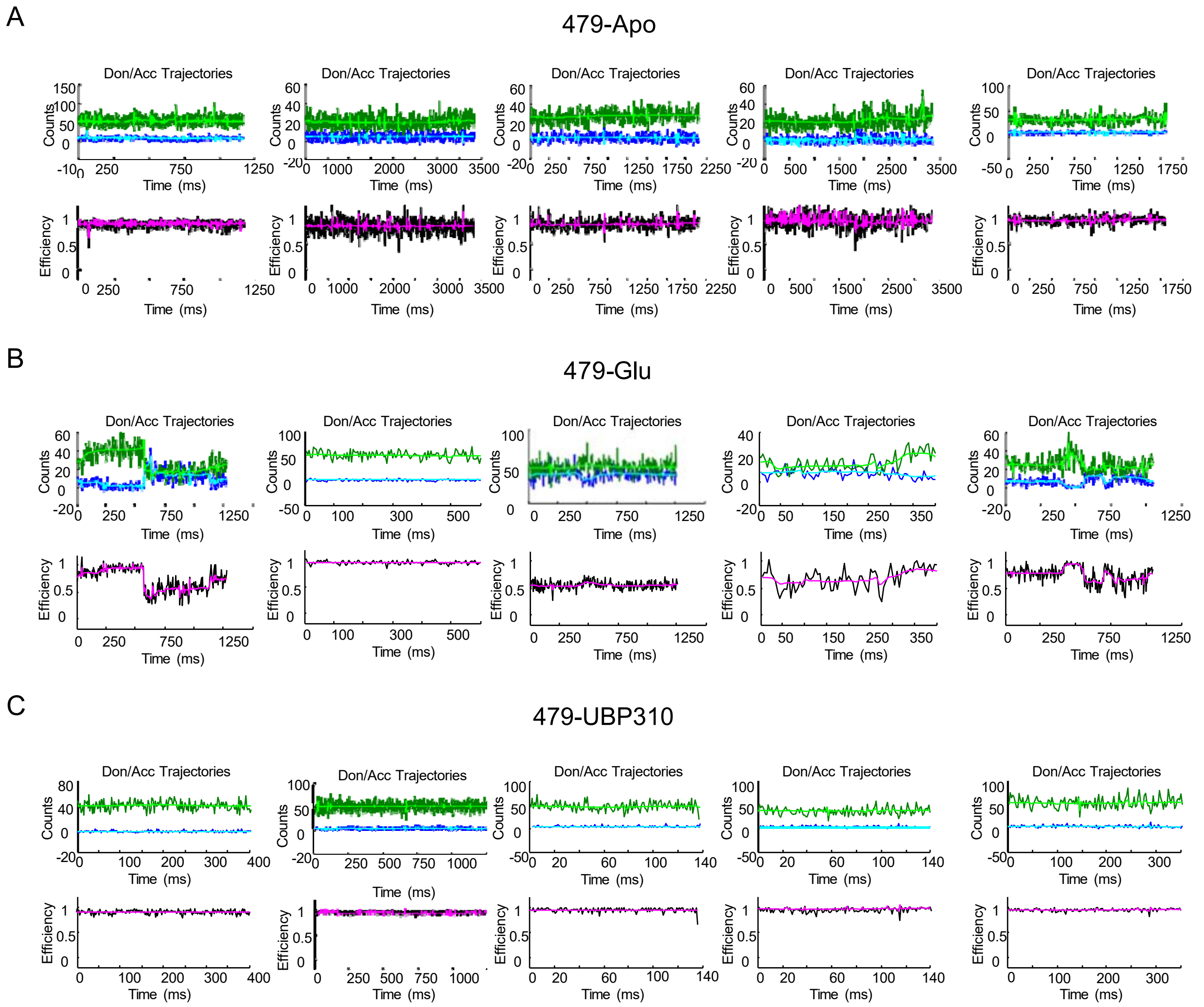

Figure S3

Representative FRET efficiency traces showing the donor (blue), acceptor (green) and FRET efficiency (black) with wavelet denoising shown in cyan, neon green, and magenta respectively, in the FRET region for site 479 in the (A) apo state, (B) glutamate-bound state, and (C) UBP310 antagonist bound states.

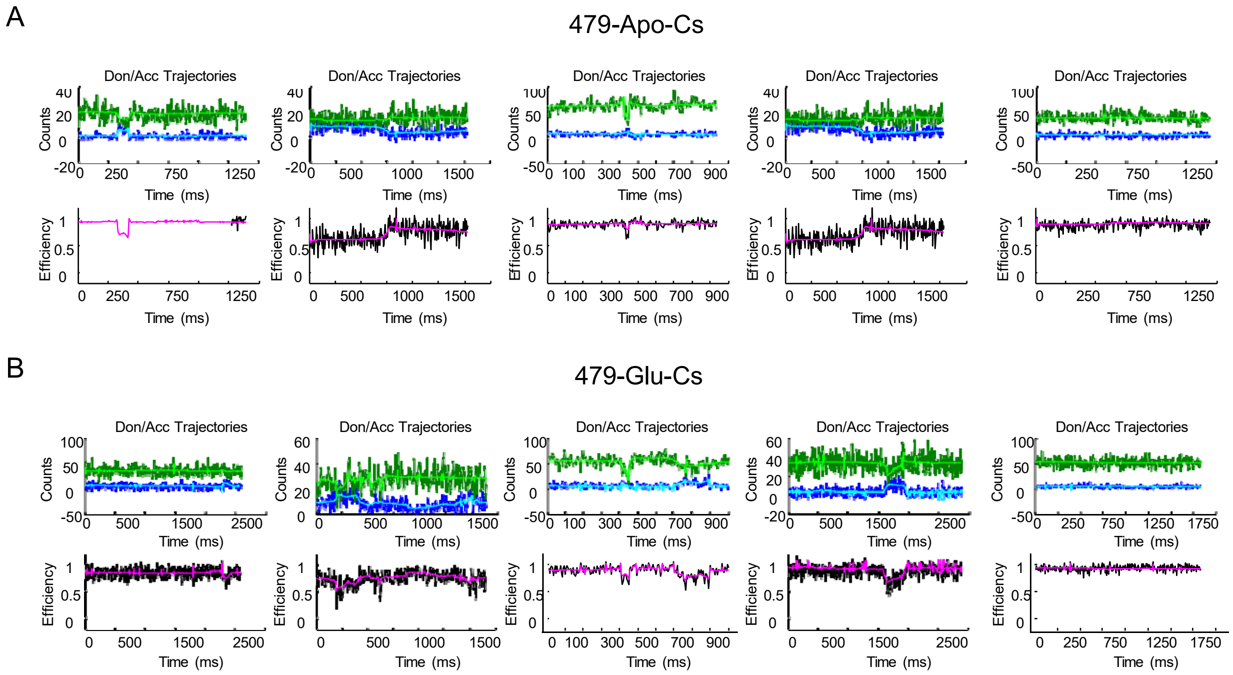

Figure S4

Representative FRET efficiency traces showing the donor (blue), acceptor (green) and FRET efficiency (black) with wavelet denoising shown in cyan, neon green, and magenta respectively, in the FRET region for site 479 in CsCl for (A) apo state and (B) glutamate-bound state.

A

479-D776K-Glu

B

479-D776K-Glu-Cs

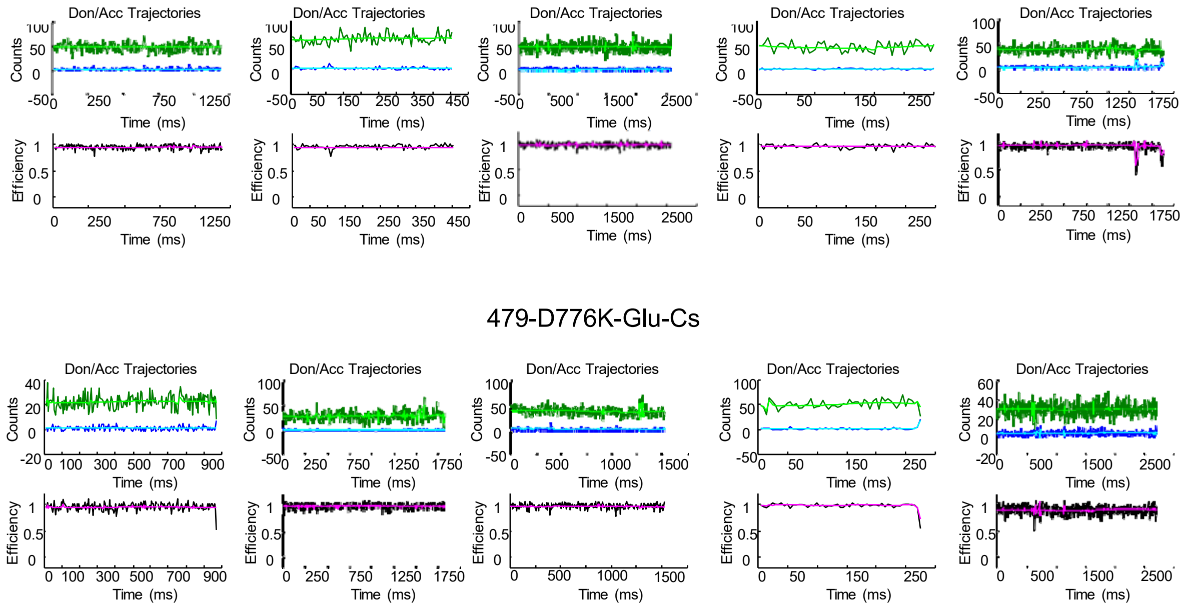

Figure S5

Representative FRET efficiency traces showing the donor (blue), acceptor (green) and FRET efficiency (black) with wavelet denoising shown in cyan, neon green, and magenta respectively, in the FRET region for site 479 in D776K background in the (A) glutamate-bound state in NaCl and (B) glutamate-bound state in CsCl.
